# The multicomponent model of working memory fifty years on

**DOI:** 10.1177/17470218241290909

**Published:** 2024-11-08

**Authors:** Graham J. Hitch, Richard J. Allen, Alan D. Baddeley

**Affiliations:** 1 Department of Psychology, University of York, York, UK; 2 School of Psychology, University of Leeds, Leeds, UK

**Keywords:** Working memory, executive control, multicomponent model, central executive

## Abstract

We provide a broad overview of our original investigation of working memory; how the multicomponent model followed from our use of a dissociative methodology; and our intention that it should be simple, robust, and applicable. We describe how subsequent development of the model has increased its scope, depth, and applications while at the same time retaining its core features. Comparisons with the growing number of alternative models suggest agreement on the basic phenomena to be explained and more similarities than differences. While differences between models attract interest, we caution that they do not necessarily reflect the most important issues for future research, which we suggest relate principally to the nature of executive control. The longevity of the multicomponent model reflects not only the importance of working memory in cognition but also the usefulness of a simple, robust framework for further theoretical development and applications.

In our initial paper (Baddeley & Hitch, 1974), we investigated whether the resources that support the limited span of short-term memory (STM) serve as a general-purpose working memory in cognition. This had been claimed several times (e.g., Atkinson & Shiffrin, 1968; Miller et al., 1960; Newell & Simon, 1972) but, so far as we could see, had not been systematically investigated. If STM does indeed act as a general-purpose working memory, we would expect this to have important practical implications. However, some of the few results available at the time seemed to go against the idea. For example, Patterson (1971) tested the hypothesis that STM is used to hold the retrieval plan in recall from long-term memory (LTM) by studying the effect of counting backwards for 20 seconds in between the recall of successive items, a procedure known to be effective in erasing the contents of STM (Peterson & Peterson, 1959). However, the results showed that disrupting STM had no effect on retrieval from LTM. The need to question the hypothesis that STM acts as a working memory was also raised by the case of a neuropsychological patient whose STM capacity, as measured by his auditory-verbal memory span, was severely reduced but who nevertheless suffered no obvious general deficit in cognition (Shallice & Warrington, 1970). It was in this somewhat unpropitious context that we set out on our original investigation.

## Origins of the multicomponent model

We set out by asking ourselves the simple question “What is STM for?” and attempting to find an answer through experiments. Our technique was to simulate the neuropsychological patient’s memory span deficit in healthy adults by loading their STM with irrelevant information and looking to see whether this had any effect on their cognition. We studied three different aspects of cognition in this way: verbal reasoning, prose comprehension, and free recall learning. The interesting finding was that the pattern of results was the same for all three activities, despite their diversity. Thus, there was moderate but far from catastrophic interference when STM capacity was filled to capacity and little or no interference when the load on STM was well below memory span. This result suggested that STM serves as only part of a working memory system that must involve something extra. We proposed the multicomponent model as a deliberately simplistic interpretation. It assumes the extra component is a limited capacity “central executive,” responsible for attention-demanding control processes necessary in many aspects of cognition. We characterised the STM component as an “articulatory loop” in which stored information decays rapidly but can be refreshed by subvocal rehearsal. We assumed that rehearsing a small memory load in this “phonological loop” (as it is now known) does not draw on the resources of the central executive and that even rehearsing a high memory load leaves executive resources largely free for other activities. The multicomponent model was immediately useful in offering a new way to interpret Shallice and Warrington’s patient, who could be regarded as suffering from selective impairment of the phonological loop while the central executive remained intact.

At the time of our investigation, STM was widely regarded as a verbal store (Atkinson & Shiffrin, 1968; Murdock, 1967; Waugh & Norman, 1965), and it was for this reason the cognitive activities we chose to investigate were primarily verbal. However, although not nearly as extensively studied as verbal STM, clear evidence of memory for spatial location existed based on both visual (Dale, 1973; Warrington & Baddeley, 1974) and tactile input (Gilson & Baddeley, 1969), while the pattern span method of testing short-term visual storage was in process of development (Phillips, 1974; Phillips & Baddeley, 1971). Meanwhile, the link between visual STM and the active use of mental imagery, originally stimulated by the work of Brooks (1967), was being actively pursued and incorporated into the developing working memory model (Baddeley, Grant et al., 1975; Baddeley & Lieberman, 1980). Hence, although the initial case for proposing a multicomponent working memory was based on data from verbal tasks, there already existed a growing body of evidence indicating the need to include at least one other component storing visuospatial information. In general, we regard the main function of buffer storage as providing a bridge between rapid streams of perceptual or motor data and more slowly changing internal representations, but it can also be used to support cognition by maintaining temporary information in activities such as mental arithmetic (Hitch, 1978) and navigation (Garden et al., 2002).

Looking back, our aims in presenting the multicomponent model were threefold. The first was to offer a robust theoretical account of working memory. The chances of achieving this were enhanced by having found similar effects of loading STM in cognitive tasks that were quite different from one another. Our second aim was to offer a minimal, deliberately incomplete model that would provide a useful basis for asking further questions and for theoretical development. Our third aim was to encourage the wider applicability of our findings by ensuring the model was easy to explain to non-experts and for them to use. Attempting to meet all these aims entailed keeping the model as simple as possible.

It is perhaps worth pointing out that the dissociative logic behind our dual-task methodology led naturally to the type of model we proposed. The multicomponent model is essentially a high-level map of the working memory system drawn to reflect the empirical dissociations we observed. The individual components reflect broad categorical distinctions we regard as analogous to the separation of continents in geography. In both cases, the distinctions are useful at a high level of description and analysis, even though lower-level details, such as exact boundaries, are not yet fully understood. In this way, the multicomponent model provides a framework for investigating processes and mechanisms further, even though we could not say a great deal about these when the model was first proposed.

While the multicomponent model has principally focused on behaviour rather than its neural basis, evidence from neuropsychology has played an important part in its development while approaches based on neurophysiological methods have become increasingly widespread and influential. Though beyond the scope of the current review, we regard such developments as important in informing theory and likely to prove practically important in understanding and treating clinical and developmental disorders.

## Developing the multicomponent model

During the last fifty years, the multicomponent model has been progressively expanded and refined to increase both its scope and its applicability. These developments have been written about extensively elsewhere (e.g., Baddeley, 1986, 2000, 2007, 2012; Baddeley et al., 2010, 2021), allowing us to skip many details here and concentrate on an overview. For ease of presentation, we have organised this in separate subsections on theoretical developments and applications. However, it is important to bear in mind substantial cross-fertilisation between theory and application throughout.

### Theoretical developments

These include more detailed accounts of buffer storage in the phonological loop (Baddeley et al., 1984; Burgess & Hitch, 1992, 1999, 2006; Page & Norris, 1998, 2009) and the visuospatial sketchpad (Baddeley, 2007; Logie, 1995). Problems with the overall model, such as the neglect of LTM and provision for integrated representations, led to a major revision and extension (Baddeley, 2000). It is convenient to organise the discussion in terms of the various components of the model.

#### Phonological loop

The phonological loop was the first component to be investigated. Evidence that immediate serial recall is poorer for words that take longer to say and the abolition of this effect by articulatory suppression confirmed the idea that information stored in the phonological loop is rapidly forgotten unless refreshed by subvocal rehearsal (Baddeley, Thomson et al., 1975). Further investigation indicated that spoken words access the phonological loop directly whereas written words do so indirectly, requiring subvocalisation (Baddeley et al., 1984). A related distinction between “the inner ear” and “the inner voice” (Baddeley & Lewis, 1981) proved useful in explaining the otherwise puzzling observation that articulatory suppression interferes with judging whether words rhyme but not whether they sound alike (Besner et al., 1981). The distinction is also useful in accounting for cases of neuropsychological impairment of auditory-verbal STM, as these can be understood in terms of selective damage to the input storage component of the phonological loop (Shallice & Papagno, 2019).

Initial evidence for an effect of the spoken duration of words on their immediate recall suggested that memory traces in the phonological loop decay rapidly in the order of 2s in the absence of rehearsal (Baddeley et al., 1975). However, subsequent research questioned whether there is an effect of word duration (e.g., Caplan & Waters, 1994; Lovatt, Avons & Masterson, 2000), and other evidence cast doubt on time-based trace decay as an account of rapid forgetting (Oberauer & Lewandowsky, 2008). These observations (see also Guitard et al., 2018) have clearly challenged our initial view of the way the phonological loop loses information. Our current position is that the issue is likely to remain unresolved until such time as we have better methods for identifying the processes that underpin short-term forgetting. In the meantime, uncertainty on this specific detail does not challenge the overall concept of the phonological loop as a limited capacity, speech-based store that loses information rapidly and can be refreshed by subvocal rehearsal (Baddeley & Hitch, 2019).

Other critics regarded the phonological loop as of little importance in cognition given how little information it can hold. However, interest in it was boosted by evidence that its bridging function is crucial in the learning of new vocabulary (Baddeley et al., 1998). The basic idea is that when first heard, a new word is stored in the phonological loop as a string of sublexical elements, and repeated exposure results in their integration into a lexical unit in LTM. We discuss its applications in a later section.

The work on vocabulary acquisition drew attention to the importance of two aspects of the phonological loop that were not specified in the original multicomponent model. One concerns its interactions with LTM, the other how it maintains information about serial order. We initially set these to one side for the sake of simplicity despite knowing the importance of chunking based on LTM in immediate recall (Miller, 1956) and the key role of order information in STM (Conrad, 1965). By good fortune, our interest in these matters was growing at a time of rapidly developing opportunities for simulating behaviour computationally, as artificial neural networks (e.g., Hinton & Anderson, 1981). Our first attempt to simulate the phonological loop focused on the problem of serial order and was based on the idea that selecting items in order involves a process of competitive queueing (Grossberg, 1987; Houghton, 1990). Our computational model reproduced the hallmark effects of the phonological loop (phonemic similarity, word length, and articulatory suppression) but went further by generating serial position curves and order errors, aspects of recall the multicomponent model had not addressed (Burgess & Hitch, 1992). Further development of the model went on to address long-term learning by adding a process for matching between a presented sequence and previously learned ordered representations stored in LTM, showing good agreement between simulations and experimental data (Burgess & Hitch, 2006; Hitch et al., 2009). In parallel, our colleagues Mike Page and Dennis Norris had been developing a broadly similar competing model (Page & Norris, 1998), and they extended it to address the limitations of the matching process we had proposed (Page & Norris, 2009).

Although computational modelling has advanced our understanding of the phonological loop, two comments stand out for mention. One is that the simple idea of the loop as a limited-capacity, speech-based store continues to retain its utility at a broader level, as when discussing its contributions to working memory and cognition. The other is that despite their sophistication, computational models of the phonological loop lack any vestige of central executive control and so need extra assumptions to capture the use of the loop in goal-driven activities such as reasoning or mental arithmetic. The value and applicability of computational models in the future will depend on the extent to which they can meet this challenge of simulating high-level supervisory control.

#### Visuospatial sketchpad

Our interest in visuospatial working memory was prompted by a series of ingenious experiments by Brooks who devised tasks that encouraged or discouraged the use of visual imagery, showing that performance interacted with the visual or verbal modality of response. Imagery helped when it mapped onto a visually based strategy (Brooks, 1967) and hindered when a visuospatial response interfered with imagery maintenance (Brooks, 1968). We adapted the Brooks imagery tasks using a dual task design in which participants were required to combine the visuospatial or verbal memory tasks with a concurrent visuospatial tracking task that involved keeping a stylus in touch with a rotating spot of light (Baddeley et al., 1975). Concurrent tracking disrupted the visuospatial but not the verbal task, suggesting a clear visuospatial component to our imagery task. This raised the further question of whether the disrupted imagery was visual, spatial, or both. Further experiments combined the Brooks tasks with a spatial but nonvisual dual task involving keeping track of a moving auditory stimulus while blindfolded or with a visual but nonspatial dual task involving judging the brightness of a light field. Our results suggested that the imagery used in our tasks was spatial rather than visual (Baddeley & Lieberman, 1980). Logie (1986), however, went on to demonstrate that a clear visual imagery component could also be shown using a different memory task. Participants were instructed to learn a spoken list of concrete nouns using either visual imagery or a rote verbal strategy. He found that visual imagery led to higher performance but was more disrupted than the verbal strategy by the concurrent presentation of nonspatial irrelevant visual stimuli such as line drawings, visual noise patterns, or even simple colour patches. It became clear that the sketchpad has separable visual and spatial components (Klauer & Zhao, 2004; Logie, 1995; Logie et al., 1990). From a different perspective, Nelson Cowan (personal comm.) has made the useful point that it is not clear why the visual and spatial components of working memory are grouped together, as in the multicomponent model. We agree with his suggestion that research on congenitally blind individuals could shed light on the possibility of a separate spatial component, and we would add to this the possibility of separable motor, tactile, and kinaesthetic components (Baddeley et al., 2011; Li et al., 2022). We note here that this potential expansion of the multicomponent model shifts its scope somewhat closer to Cowan’s (1988) embedded process model, a topic we revisit later.

A second stream of research has focused on how visuospatial information is retained in the short term. An ingenious study by Posner and Keele (1967) analysed retention of the visual appearance and names of letters following a range of brief delays, measuring both reaction time and errors, concluding that the visual characteristics survived for about 1.5 seconds while the verbal representation was more durable. An alternative explanation of their results, however, was proposed by Phillips and Baddeley (1971). They suggested that both name and visual pattern-based codes were operative, with the verbal being slower but then dominating the visual. To test the idea of a more durable visual pattern code, Phillips and Baddeley (1971) employed a task using non-nameable stimuli comprising 5x5 matrices of which half the cells were filled. Retention was tested after delays of up to 9 seconds by presenting either the same or a second matrix in which a single cell was changed. The pattern of both errors and reaction times resembled that found for the Posner and Keele verbal data, demonstrating a more durable trace than that proposed by Posner and Keele. Phillips (1974) went on to apply the change detection method in a series of further experiments showing that larger matrices showed greater forgetting, with results indicating contributions from both brief sensory storage and a later more durable short-term storage system, while further work demonstrated a recency effect when more than one matrix was presented (Phillips & Christie, 1977).

The change detection method was then used by Luck and Vogel (1997) in a classic series of studies showing that features requiring separate perceptual processing channels, such as colour, shape, and location, when combined to form coloured shapes, could be remembered as well as the individual features, with a limit of about four items. This suggested that visuospatial working memory is primarily object-based, though subsequent evidence has indicated that features can be individually lost when complexity increases (Hardman & Cowan, 2015; Oberauer & Eichenberger, 2013). The study of visuospatial working memory has expanded rapidly in recent years, combining techniques from the fields of vision and attention, using methods drawn from both psychology and neuroscience. These have been applied to address questions concerning the structure and limits of visuospatial working memory, including how many features can be bound into a single object, the extent to which capacity is feature or object based, and how continuous features such as a range of different colour shades might be stored or lost (e.g., Adam et al., 2017; Ma et al., 2014; Oberauer, 2022; Wheeler & Treisman, 2002; Zhang & Luck, 2008). Our feeling is that its capacity likely reflects limits on both features and objects (e.g., Forsberg et al., 2023), depending on the task and materials, and that time and spatial location play a role in supporting such representations (Karlsen et al., 2010; Schneegans et al., 2023). Though we remain agnostic on the continuing question of whether capacity reflects a limited number of slots or a set of resources, meaningful performance capacity is clearly functionally limited to a few items.

Our own approach has principally been to apply methods that have already proved useful in studying verbal working memory to its visuospatial equivalent, including dual-task and perceptual interference applied to paradigms adapted from the visual literature (Luck & Vogel, 1997; Wheeler & Treisman, 2002). These studies have generally been carried out in the context of our interest in the episodic buffer component of the model so are discussed in more detail in that section, but they broadly indicate that storage of visual features and objects draws on the central executive and is subject to endogenous strategic control and the relatively automatic influence of exogenous perceptual input (see Allen et al., 2024; Baddeley et al., 2011; Hitch et al., 2020, for reviews). What and how much is remembered will reflect the current memory load in relation to the individual’s ability, the strategies that can be usefully applied, and the flexible distribution of limited attentional control resources across the item set.

#### Central executive

The central executive was a novel concept about which little was said initially beyond the claim that its limited capacity resources could be allocated flexibly between control processes and temporary storage (Baddeley & Hitch, 1974). As such, it was wide open to criticism for being little more than a label for the homunculus (e.g., Parkin, 1998). In its defence, however, it did succeed in emphasising the importance of internally driven attentional control processes in working memory and, in so doing, setting the agenda for further investigation.

Early studies of dual-task performance gave little support for the proposed trade-off between resources for processing and storage in the central executive. A better view of how it operates was eventually inspired by a model of the control of thought and action, which assumes competing action schemata are activated by their respective calling cues (Norman & Shallice, 1986). In familiar situations, well-learned schemata compete for selection automatically and can avoid conflict without the need for conscious attentional control. However, when automatic selection fails or is not possible, for example, in a novel situation, a limited capacity supervisory attentional system is called into play, biasing activation levels and monitoring success. Thinking about the central executive in these terms, as a purely attentional resource, proved useful in accounting for patterns of dual-task interference that were previously difficult to explain (Baddeley, 1986). We discuss the nature of the central executive in more detail in a later section.

An unforeseen consequence of viewing the central executive as an attentional resource was to leave the phonological loop and visuospatial sketchpad as the only buffers storing temporary information, and it gradually became clear this was problematic (Baddeley, 2000). For example, the immediate recall of a series of words vastly exceeds the capacity of the phonological loop when they form a meaningful sentence (Brener, 1940). This suggests some form of integrated storage, in this case drawing on LTM. Miller’s (1956) famous illustrations of chunking in STM make the same point. Similarly, the observation of the effects of visual similarity in the immediate recall of verbal material (Logie et al., 2000) suggested a form of storage that integrates across the phonological loop and the visuospatial sketchpad. More generally, the everyday experience of creating and manipulating multimodal mental models emphasises the importance of integrated representations in working memory. These considerations led to a major extension of the multicomponent model to include an episodic buffer, seen as a limited capacity store for integrated, multimodal representations, accessed by conscious awareness (Baddeley, 2000). It is perhaps worth noting that the seeds of the episodic buffer were already present in the 1974 chapter with its assumption that the central executive had a storage function, a function that was for some time omitted and ignored (Baddeley, 1986) until its eventual reappearance in the form of the episodic buffer. Also included in the extended model for greater completeness was the long overdue addition of LTM, with separate verbal, visuospatial, and episodic components interconnecting with their corresponding buffer stores.

#### Episodic buffer

The extension of the multicomponent model led us to become interested in the binding processes whereby different sources of information combine to form integrated representations in the episodic buffer. We began by testing the initial assumption that binding processes draw on the central executive using dual-task studies of immediate memory for a small set of coloured shapes in which we compared memory for colour-shape combinations with memory for colours or shapes alone. We expected that an attentionally demanding concurrent task would disrupt memory for bindings more than memory for individual features. The results showed our assumption was wrong, suggesting that binding is not especially dependent on the central executive (Allen et al., 2006, 2012). Given that performance levels are typically poorer for bindings than for features, it has been claimed that the *proportional* dual-task cost is nevertheless higher for bindings (Cowan, Bao et al., 2024, p. 190). However, we found no support for this possibility when we reexamined our data as a proportional dual-task cost.^1^ To examine whether this result extended beyond the binding of features within perceptually integrated objects, we carried out follow-up studies in which shape and colour were presented in separate spatial locations or at different points in time, or one verbally and the other visually (Karlsen et al., 2010). In each case, an attentionally demanding concurrent task disrupted memory for bindings no more than individual features. Parallel studies in the verbal domain comparing structured and unstructured word lists gave the same result (Baddeley et al., 2009). The consistency of these findings led us to conclude that initial binding processes during encoding are largely automatic, and consequently, the episodic buffer is a more passive store than we had initially supposed, at least for the kinds of stimuli and binding decisions investigated in these studies. This then led us to adjust the extended model by allowing all subsystems direct access to the episodic buffer rather than accessing it solely via the central executive (Baddeley et al., 2010, 2011).

Different principles may apply to maintenance, however, and an incidental finding gave useful information about the short-term storage of feature bindings. It came from a study in which a sequence of coloured shapes was immediately followed by a probe testing recognition memory for one of the feature bindings or individual features (Allen et al., 2006, Experiment 5). There was a through-list recency effect in both conditions with excellent memory for the last item, consistent with previous research (Phillips & Christie, 1977; Walker et al., 1993). However, the recency gradient was steeper for bindings than features (see also Allen et al., 2014, 2017), a new finding suggesting items are encoded as integrated representations, and these subsequently fragment into their separate features (cf. Jones, 1976). We assumed that the contents of the episodic buffer reflect the focus of attention at any instant in the form of an integrated object file (Treisman, 1986) and that recently attended items are represented in the visuospatial sketchpad in various states of fragmentation. Converging evidence for this account came from the effect of loading the central executive with the concurrent task of counting backwards, which impaired memory for all except the most recently presented item (Allen et al., 2014). Thus, paying perceptual attention to items during a presentation is not highly demanding of executive resources, but these resources are heavily engaged in maintaining items undergoing fragmentation in-store via attentional refreshing (Barrouillet & Camos, 2015).

A different strand of research suggested that the role of perceptual attention could be explored further by studying the effect of a post-stimulus perceptual distractor on retention. We began by studying memory for a simultaneous display of coloured shapes. The distractor, or “stimulus suffix,” was a coloured shape drawn from either the same feature space as the memory items or a different, perceptually distinct feature space. Results suggested two components of suffix interference—one affecting memory for features and bindings to the same extent, the other having a specific effect on memory for bindings, especially when suffix and memory items were drawn from the same feature space (Ueno, Allen et al., 2011). Further experiments using cued recall showed that a suffix from the same feature space induced intrusion errors in recalling a feature of the suffix (Ueno, Mate et al., 2011). We concluded that a distracting stimulus that matches the task set for memorisation tends to draw perceptual attention and thereby gain access to the episodic buffer.

Our next experiments took the obvious step of examining the effect of a post-stimulus suffix distractor on memory for a series of coloured shapes, which would allow us to contrast memory for the most recent and earlier items. Using cued recall once more, we found that a suffix interfered with memory for the most recent item much more than earlier items and that the effect was more pronounced when the suffix was drawn from the same feature space as the memorised items (Hu et al., 2014; Exp. 1). This reinforced our view that the episodic buffer holds a bound representation of each item in a series in turn, as it occupies the focus of perceptual attention. However, we soon realised this view was too simple, having noticed that our participants occasionally reported concentrating on memorising the first item in a series, even though all items had an equal chance of being tested. This observation led us to investigate strategies by giving instructions on how to prioritise items differently. In one condition, participants were told correct recall of the first item would earn a bigger reward than would the other items, and in a mirror condition, the item presented last earned the bigger reward (Hu et al., 2014, Exp. 4). The effect was to boost memory for the high-priority item and reduce memory for low-priority items, leaving the overall amount of information unchanged, a trade-off that has been replicated in subsequent work (e.g., Atkinson et al., 2018; Hitch et al., 2018; Hu et al., 2016, 2023). The multicomponent model offers a ready explanation of the trade-off in that the central executive has a top-down influence on the way storage capacity is utilised in our experimental task without affecting storage capacity per se (Hu et al., 2016). A review of the growing body of research on prioritisation effects (Allen et al., 2024) shows our basic findings are robust and generalise across memory tasks involving a range of different types of information (e.g. Atkinson et al., 2021, 2022, 2024; Roe et al., 2024).

One explanation of our results is that prioritisation alters the schedule of attentional refreshing such that a high-priority item is represented in the episodic buffer for longer or more often. We explored this possibility by examining the effect of a perceptual distractor (suffix) when either the first or last item in a series was prioritised. When the final item was prioritised, results were broadly the same as in the absence of the instruction, in that presentation of a suffix interfered with memory for the final item most and did so more when it was drawn from the same feature space as the memorised items (Hu et al., 2014). However, when the first item was prioritised, suffix effects were observed for the first as well as the final item and varied with the features of the suffix in the same way in each case. Similar findings have been reported in subsequent studies (Allen & Ueno, 2018; Hitch et al., 2018; Hu et al., 2016), though we note that they do not always reliably generalise with changes to task context (Hu et al., 2023; Vergauwe et al., 2023). Nevertheless, the apparent effect of prioritising the first item on its susceptibility to perceptual interference is consistent with our view that prioritisation affects the schedule of attentional refreshing, such that a high-value item is more likely to occupy the episodic buffer at any point in time.

The episodic buffer has, therefore, proved a productive concept in motivating research on feature binding, automatic perceptual input, and strategic attentional control. It also offers a way for the multicomponent approach to broadly capture the complexity of multi-domain and -modality tasks where different input streams are available or required for performance. This includes tasks designed to directly assess binding across modalities such as vision and audition (Allen et al., 2009; Wang et al., 2015) and vision and olfaction (Johnson & Allen, 2023). Similarly, it allows the model to incorporate findings showing enhanced working memory performance when multiple forms of representational code (e.g., verbal, visuospatial, and motoric) are available, as in memory for instructions (Allen et al., 2023) and visuospatial bootstrapping of verbal material such as digits (Darling et al., 2017). In the latter case, prior knowledge in LTM also appears to play a role in that the effect is generally limited to familiar configurations (see also the advantage for structured sentences over word lists; Allen et al., 2018; Baddeley et al., 2009). Thus, the episodic buffer provides a way of pulling together different forms of processing and prior knowledge into a consciously accessible, multidimensional form.

To summarise, our results support a view that equates the episodic buffer with the current focus of attention. We see this as comprising a limited number of integrated, bound representations, their identity influenced by both perceptual and internal control processes, the latter reflecting task set in the form of goals and plans. These external and internal influences can be in alignment—for example, when a single item is presented for immediate recall. However, more generally, perceptual encoding and internal control require coordination, as when refreshing early items during the encoding of later items in a series (Allen et al., 2014; Barrouillet & Camos, 2015). Coordination is also presumably necessary when the shape and colour of a memorised object are presented at different points in time, in different spatial locations or in different modalities, even though these situations seem to place a minimal load on the central executive (Karlsen et al., 2010). Last, but by no means least, the combination of perceptual encoding and internal control can generate conflict, as when perceptual attention is paid to a suffix distractor containing features that match the task set for stimuli to be remembered, disrupting their retention. The picture of the episodic buffer to emerge is one of a dynamic, limited capacity, multimodal store that operates at the interface between external and internal attention. However, it is important to recognise that the experiments we discuss here provide only a glimpse of the episodic buffer in the relatively simple context of immediate memory and that generalisation is an obvious issue. In this context, we note wider debate as to whether the focus of attention can extend beyond the one or two items we have inferred from our own experiments (Gilchrist & Cowan, 2011). More generally, there is clearly much yet to discover by studying the episodic buffer more widely in cognition and behaviour where perceptual attention and internal control processes align, coordinate, and compete in a greater variety of ways.

### An illustration of our current view

**Figure 1.**
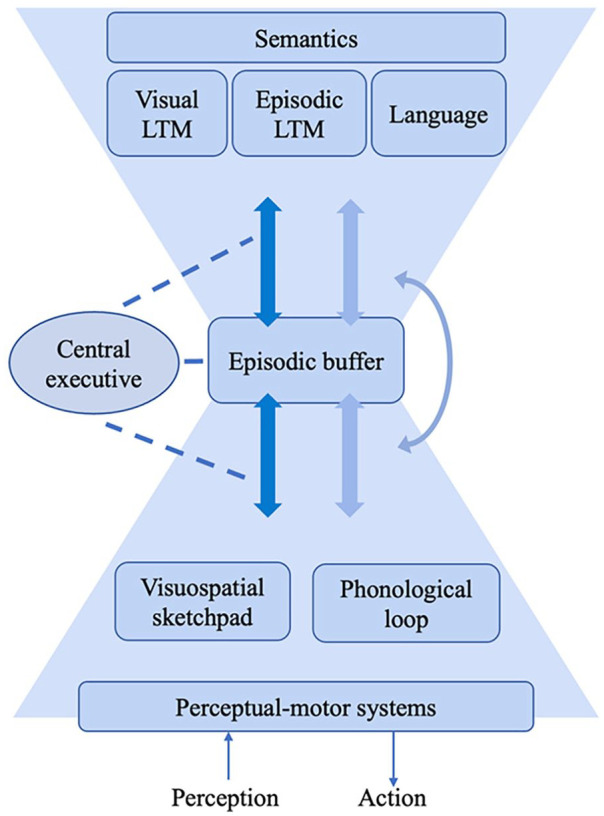
A multicomponent view of the cognitive system with working memory located between semantic long-term memory and perceptuo-motor subsystems with the episodic buffer as the central hub. The central executive is shown as a separate resource that can be deployed in a variety of ways to interact with many parts of the system. Light and dark arrows indicate implicit and explicit processes, respectively.

At this point, it will be useful to present the multicomponent model as we currently see it, setting working memory in the context of perceptual input, action, and LTM. Figure 1 illustrates our current view. It has the same components as the earlier major revision (Baddeley, 2000) but differs principally with respect to the central executive and the episodic buffer. We now locate the episodic buffer as a central hub. This follows from our work suggesting it functions as the focus of attention, at the point of interaction between internal executive control and externally driven attentional demands. We see the central executive as a separate set of control resources that can interact with the rest of the system in many ways. It can, for example, help keep information active in the episodic buffer and can also pull in information from specialised systems and LTM, as illustrated by the dark-blue arrows. Lighter arrows indicate the automatic transmission of information. It is important to emphasise that automatic processes can become controlled, as when responding to an unexpected event, and that controlled processes can become automatic, as when they become highly familiar and routine. The operation of the central executive is discussed in more detail in a later section.

### Applications

One of the main aims of creating the multicomponent model was that it should, in its basic form, be simple and clear enough to be applied outside the laboratory to a wide range of practical problems. Evidence that this has been the case is cited by Byrnes and Miller-Cotto (2023) who list several areas of application; in each case, we cite just one paper providing access to the relevant literature. They are (1) academic skill development (Peng, Barnes et al., 2018), (2) native language development (Baddeley et al., 1998), (3) foreign language learning (Linck et al., 2014), (4) learning disability (Peng, Wang et al., 2018), (5) attention deficit hyperactivity disorder (Willoughby et al., 2019), and (6) ageing (Nguyen et al., 2019). With the exception of the review by Baddeley et al. (1998), all these are relatively recent applications of the model by groups with no direct connection with ourselves, which we regard as encouraging. To give a more detailed flavour of our own contribution to the application, we have chosen three different areas—namely language acquisition, mathematics, and emotion.

#### Language acquisition

There is by now a very extensive literature on working memory and language as reflected in the recent *Cambridge Handbook* (Schwieter & Wen, 2022), comprising some 40 chapters from contributors across a range of disciplines, not all of course involving the multicomponent model. For present purposes we will focus on the role of the phonological loop for which Papagno (2022) provides an update of work concerned with the proposal that the loop acts as a language-learning device, indicating the substantial replicability of the principal claims on which the proposal was based. She cites some 10 studies indicating the role of the phonological loop in native language development with a pattern suggesting that the phonological loop drives the rate of new word learning in the earlier stages while existing word knowledge becomes increasingly important as vocabulary grows (Gathercole et al., 1992; Papagno, 2022). Much of this work has used the Nonword Repetition Test in which the child attempts to repeat back pseudo-words increasing in syllabic length, providing a closer analogue to new word acquisition than digit span. This has also been shown to be a useful marker of a range of language disabilities, including specific language impairment (Gathercole & Baddeley, 1990), dyslexia (Baddeley et al., 1988), and Down syndrome (Jarrold et al., 1999), while a phonological loop deficit has been found to characterise a number of neuropsychological conditions ranging from classic cases of verbal STM dysfunction (Shallice & Warrington, 1970; Vallar & Baddeley, 1984) to dysarthria, reflecting disruption of the articulatory system (Baddeley & Wilson, 1985; Carlesimo et al., 2006). The phonological loop also plays an important role in second-language learning in both children and adults with early studies (Atkins & Baddeley, 1998; Service, 1992), shown to be widely replicable by Linck et al. (2014), whose meta-analysis of 79 samples involving over 3000 second-language learners showed substantial separable contributions from both executive and phonological aspects of working memory.

#### Mental arithmetic

A typical multidigit computation involves a series of simpler stages that generate partial results, and when performed mentally, these must be stored over brief intervals to obtain the solution (Hitch, 1978). It, therefore, provides a natural example of information processing in combination with temporary information storage and an obvious opportunity to apply the multicomponent model. There have by now been many such applications, typically involving dual-task methodology in which mental calculation is accompanied by a secondary task designed to tap the central executive, phonological loop, or visuospatial sketchpad (the episodic buffer requires more subtle methods and has not so far been investigated in this context). Reviews of dual-task interference indicate that mental arithmetic depends primarily on the central executive and phonological loop, with rather less certainty about the visuospatial sketchpad (see, e.g., Chen & Bailey, 2021; DeStefano & LeFevre, 2004; Raghubar et al., 2010). Detailed investigations suggest that the executive and the loop are more heavily loaded when operations such as carrying or borrowing are required, consistent with the controlling function of the former and the storage function of the latter (Fürst & Hitch, 2000; Imbo et al., 2005). The involvement of the sketchpad depends on task features such as whether operands are presented visually or orally (Logie et al., 1994) and whether they are arranged vertically or horizontally (Trbovich & LeFevre, 2003), though some studies have reported negative results (e.g., Noel et al., 2001; Seitz & Schumann-Hengsteler, 2000). There are also interesting cross-cultural differences, one notable example being the effects of training to calculate using an abacus, a manual device that represents numbers as spatial patterns using movable beads on fixed rods. People trained this way report using a “mental abacus” when performing mental calculations (Hatano et al., 1977; Stigler, 1984; see also Frank & Barner, 2012), suggesting a different role for the visuospatial sketchpad across cultures.

In addition to experimental studies of mental calculation in adult populations, the multicomponent model has been widely used in more general studies of schoolchildren’s arithmetic. A typical study shows that individual components of the model can be used to predict individual differences in children’s arithmetical abilities (e.g., Bull et al., 1999; Rasmussen & Bisanz, 2005) and a related approach assesses components of the model to help understand difficulties in children’s acquisition of arithmetical skills (e.g., Friso-Van den Bos et al., 2013; Hitch & McCauley, 1991; Holmes & Adams, 2007; McLean & Hitch, 1999).

#### Clinical applications: emotion and the episodic buffer

In exploring the nature of the visuospatial sketchpad, Idzikowski and Baddeley pursued an earlier suggestion by Hebb that eye movements might play a role in maintaining visual imagery, finding that side-to-side eye movements did indeed interfere with the use of a visual imagery–based STM task (Baddeley, 1986, pp. 115–120; Postle et al., 2006). This became clinically relevant in connection with the development of a method of treating the disturbing flashback memories associated with PTSD (post-traumatic stress disorder) whereby the patient was encouraged to follow a target such as the therapist’s moving finger while visualising the stressful event. This method, known as EMDR (eye movement desensitisation retraining), was shown to be effective but had no generally accepted interpretation. This led Andrade, Kavanagh, and Baddeley (1997) to suggest and test an interpretation in terms of working memory, proposing that the saccadic eye movements involved in EMDR may operate by reducing the vividness and emotional intensity of negative flashbacks, gradually over repeated sessions reducing their emotional impact. We went on to carry out a series of experiments exploring the impact of concurrent eye movements on forming visual images of pleasant or unpleasant scenes. Initial results based on presented stimulus items suggested a reduction in reported vividness but not emotionality. However, when we asked people to generate emotional scenes from their own experiences rather than pictures or descriptions, both vividness and emotionality were reduced. We also found disruption from a spatial tapping task, as subsequently did Holmes et al. (2004), while similar effects have been shown from other visuospatial tasks such as modelling shapes in plasticine clay (Andrade et al., 2012; Stuart et al., 2006), showing that imagery suppression can be achieved without the need for explicit eye movements.

This indeed has proved to be the case. Emily Holmes and colleagues have successfully used a popular computer game Tetris, in which the player attempts to control descending cubes and pack them into layers, as an effective imagery suppression method. In one study (Holmes et al., 2010), participants viewed a traumatic film involving death and injury, immediately followed by a 10-minute session of either Tetris, a computer-based quiz, or an unfilled rest. Holmes et al. found fewer emotional, spontaneous flashback memories in the Tetris group, an effect that lasted for up to 4 hours. This raised the question of whether such a transient effect could be amplified. Here, they took note of research on the neuroscience of memory consolidation, which indicated that when a memory has just been retrieved, it is particularly vulnerable to disruption. Could the interval immediately following the retrieval of a stressful memory be used to reduce its strength? Holmes et al. (2009) tested this by following a stressful film with a 30-second pause followed by a 10-minute session of Tetris or by an unfilled control period. Participants were asked to record flashbacks over the following week. The Tetris condition was indeed effective, providing what they refer to as a potential “cognitive vaccine”, reducing the impact of flashbacks and paving the way for its clinical application (Kessler et al., 2018).

In an extensive series of studies, Andrade, Kavanagh, and colleagues have used the working memory model to extend the work on flashbacks in PTSD to the important clinical problems associated with addiction that have in common the experience of “craving”. This is a state of mind that is powerful, difficult to resist and plays a crucial role in clinical problems ranging from smoking and alcoholism to obesity and gambling (Andrade, 2023). Much of their work reflects the observation that the emotional content of visual and auditory verbal imagery differs, with visual imagery being particularly strongly linked to emotion (Bywaters et al., 2004; Kemps & Tiggemann, 2007). The state of a heavy smoker’s craving for a cigarette, for example, tends to have a strong visual association with the image of the cigarette, which is then elaborated within working memory to evoke other features such as smell and taste. The experience is initially rewarding but short-lived unless gratified or refreshed, and then revisited and, unless rewarded by the cigarette, leads to frustration. The multidimensional image itself competes for working memory capacity with other goals—both immediate, for example understanding a revision text being read, and long term, such as graduating with a good degree. The image thus becomes both frustrating and distracting.

This approach to understanding craving, which they term elaborated intrusion theory, (Kavanagh et al., 2005; May et al., 2004) has been developed into a method of dealing with disruptive craving, termed functional imagery training. It incorporates two features, the emotional power of imagery and the observation that familiar images are more vivid than novel. The method then involves training the participant to select and maintain images associated with the desired goal, in the case of the revision example, the satisfaction of completing the assignment successfully, aiming to make the image as vivid and accessible as possible and then using it to suppress the competing image of smoking. This approach has already proved effective—for example, in inducing sustained weight loss in the treatment of obesity (Solbrig et al., 2019) and improving resilience and success in sports performance (Rhodes et al., 2018, 2020).

## Alternative models of working memory

Developments in the multicomponent model have taken place in the context of a rapid growth of interest in working memory more generally and a remarkable proliferation of alternative theoretical accounts. The first point to make is that there is substantial agreement on the key properties of working memory that all high-level models need to acknowledge and explain. This is important for any field of research and is nicely illustrated by the existence of a set of benchmark empirical observations (Oberauer, Lewandowsky et al., 2018) and a recent analysis that identifies 5 features of working memory common to all the main theoretical accounts (Byrnes & Miller-Cotto, 2023). Briefly, these are (i) limited capacity, (ii) combining processing and storage, (iii) attention playing a central role, (iv) rapid forgetting of unattended information, and (v) facilitation and interference from LTM. The original multicomponent model embraced the first 4 of these features, reflecting its intentional incompleteness, and the fifth was addressed later when the model was revised and expanded to increase its scope (Baddeley, 2000).

Alternative models differ mainly in how they seek to explain the core properties of working memory. The main proponents describe their accounts alongside one another in a recent volume edited by Logie, Camos, and Cowan (2021). Some can be seen to be broadly like the multicomponent model and to differ in details and emphasis rather than major substance. We regard the models proposed by Barrouillet and Camos (2021), Logie (2011), and Vandierendonck (2021) as in this class as they all include a capacity for attentional control processes and buffer stores prone to rapid forgetting. Engle’s model (Engle, 2018; Mashburn et al., 2021) is another account of this type but is much simpler, as it interprets working memory primarily in terms of resources for attentional control and says much less about temporary information storage.

One of the more distinctive alternative accounts is Cowan’s highly cited embedded processes model (Cowan, 1988; 1999; Cowan et al., 2021), which, it is worth noting, originated with different aims from the multicomponent model. Thus, our goal in 1974 had been to present a minimal, incomplete account of the results of our own investigation, one that could be extended in the light of further research. In contrast, Cowan’s (1988) concern was with the difficulties encountered by existing models of attention, memory, and information processing. These led him to propose a theoretical framework consistent with a wider range of available evidence than we had initially considered, a fact that goes some way towards explaining similarities and differences between the two models. In this context, it is interesting to note that the subsequent expansion of the scope of the multicomponent model (Baddeley, 2000; Baddeley et al., 2021) has resulted in the two frameworks looking more alike, though with some key differences.

The most notable similarities between the embedded processes model and the current multicomponent model are their assumption of a central executive that is closely linked to a separate multimodal focus of attention (which we have recently mapped on to the episodic buffer, e.g., Hitch et al., 2020). We have also acknowledged the importance of considering other types of information in addition to the verbal and visuospatial. The most notable difference is that the embedded processes model views STM in terms of the temporary activation of regions in LTM rather than separate buffer storage. Our original decision to assume buffer storage was heavily influenced by the evidence that STM could be selectively impaired while LTM remained intact (Shallice & Warrington, 1970), a dissociation that has been considered to present a difficulty for the embedded processes model (Norris, 2017). Another challenge for Cowan’s view of short-term storage as activated LTM is explaining the capacity to learn novel information, for example, in vocabulary acquisition. This ability cannot be reduced to the temporary activation of previously stored knowledge (Baddeley et al., 2019; Norris, 2017), as illustrated by our neural network model of verbal sequence learning, which finds it necessary to add an extra mechanism for storing novel orderings of activated representations in LTM (Burgess & Hitch, 2006). Norris (2017) gives many more reasons for assuming that STM and LTM are separate systems. Cowan (2019) has responded to these at length. He accepts the need for a mechanism capable of encoding novel associations but suggests it may be a general feature of the system for learning and memory rather than specific to STM. In response, Norris (2019) observes that the mechanism Cowan (2019) proposes looks very much like a separate STM.

Oberauer’s (2021) roadmap for a theory of working memory also views temporary storage as reflecting activated LTM and sees progress as moving from such high-level metaphors to identifying specific mechanisms. His preferred method involves developing detailed models of specific tasks and using them to generate strong, testable predictions, in the “hope we will be able to develop more comprehensive models by building on what existing narrow models have in common” (Oberauer, 2021, p. 144). We agree about the value of modelling specific tasks in detail (Burgess & Hitch, 1992, 1999, 2006) and the possibility of building on such models when enough is known to identify common computational principles for inclusion in a more general model (Hurlstone et al., 2014; Hurlstone & Hitch, 2015; 2018). In our experience, however, this is a hard, slow path to follow, and we have made faster progress by using the high-level multicomponent model to guide research in parallel, modifying it when necessary to accommodate outcomes (Baddeley, 2000; Baddeley et al., 2011, 2021).

In sum, our brief review suggests general agreement on the broad characteristics of WM (Byrnes & Miller-Cotto, 2023). Furthermore, most theoretical accounts can be seen to share core features with the multicomponent model and to differ largely in matters of detail and emphasis. The two most highly influential and contrasting approaches to ours are those of Cowan and Oberauer in which WM is viewed as activated long-term memory with no assumption of modality-specific short-term buffer stores. These two differ from one another through Cowan’s emphasis on presenting a high-level overview of the system for memory and attention versus Oberauer’s essentially bottom-up approach to theory development, with each differing from our own midway approach of expanding the scope of our initially minimalist high-level model through a combination of top-down and bottom-up changes.

## Future development

Before discussing possible future developments, it is useful to comment on a recent attempt to make progress by bringing together investigators holding different theoretical positions in a novel form of extended adversarial collaboration (Cowan et al., 2020). This involved the proponents predicting the results of crucial experiments in advance of data collection and then using the results to decide among the theories. The many strengths of this approach include requiring researchers to consider others’ models in depth to identify key differences and agree on experimental tests. However, agreeing on crucial experiments involves satisfying multiple constraints, and this can introduce its own complications, for example, in methodology. The results of the adversarial collaboration proved somewhat inconclusive, though served to emphasise and encourage greater consideration of the commonalities between positions (Cowan et al., 2020; Logie, 2023). Such an outcome is not particularly surprising given that the models were devised to explain a common set of core phenomena (Byrnes & Miller-Cotto, 2023) and as a result are similar in many respects, despite looking quite different. The increasing similarity between the embedded process and multicomponent models noted in the preceding section provides another illustration of this general point. However, while it is clearly important to resolve differences between existing models, there is a danger that focusing on such differences may be at the cost of pursuing other, potentially more fruitful ways of developing our understanding of working memory. These concern outstanding questions and wider general issues that are not adequately addressed by any current model.

The wider issue we regard as the most important concerns the nature of the central executive. It has been suggested it is time for the executive to be retired and replaced with a set of distributed processors that operate without the need for central control, in so doing avoiding the problem of the homunculus (Logie, 2016). Our own position is that this would be premature as it is far from clear how such a system would fulfil the job specification of the central executive. We see this as embracing aspects of perception, cognition, emotion, and action. Executive functions in perception include alerting, engaging, disengaging, focusing, spreading, and zooming attention; in cognition, they include planning, coordinating, switching, updating, refreshing, monitoring, and reacting; in emotion, they include monitoring, activating, inhibiting, and reacting; and in the control of action, they include setting and monitoring goals, initiating, coordinating, inhibiting, and updating. We acknowledge the overlap between the functions in each of these lists, which are presented simply to give an intuitive idea of the richness and diversity of executive functions that Logie’s emergent processes will need to perform. Few of them have yet been adequately researched, and we do not claim that they are definitive. Despite this diversity, the central executive operates as a unified, limited-capacity resource as reflected by its association with conscious awareness. In this context, it is important to note that executive processes are conscious and explicit and that the same processes can sometimes be performed automatically and implicitly without conscious awareness (see Schacter, 1992; Dienes & Perner, 1999, for further discussion of the explicit/implicit distinction). We encourage job applications for the vacant post of central executive.

As we mentioned earlier, Norman & Shallice’s (1986) model of supervisory attentional control had a major influence on our thinking about the central executive. However, given the wide range of executive functions we have discussed here, it can be seen as having been concerned mainly with the control of thought and action and saying much less about perception and emotion. A broader framework that covers the full range of executive functions would provide a more comprehensive guide to future experimentation and practical applications. It would offer a high-level view of a unified, limited capacity central executive capable of serving the numerous diverse functions in its job description, a view simple enough to apply to practical problems. Although we are unable yet to propose such a framework, we are working on it, and we encourage others to do so too given the importance of the problem. So far, we have found a potentially useful analogy with real-world systems for the management of complex, multifaceted organisations. In such systems, routine operations are delegated to free up central management capacity but, importantly, they can be elevated to central management when circumstances require, such as an unexpected event. The operation of a national government provides a useful illustration in that it must satisfy broad goals and have access to a range of functions for achieving them but has finite resources for central management operations. This is due to the time such operations take up and the limited number that can be handled simultaneously, features we regard as hallmarks of the central executive. A further key feature in our current thinking is the close link between the central executive and the focus of attention in the episodic buffer. In terms of the present analogy, we would see this as corresponding to a display of the current situation with which central management is concerned. However, all analogies have their limitations, and the most salient here is how to explain limited attention and awareness and responsiveness to events in strictly mechanistic terms, without invoking the spectre of the homunculus. There is nevertheless a clear opportunity for progress by increasing our understanding of the functions and mechanisms of the central executive and thereby reducing and clarifying what is left for a homunculus to do. In this respect, we regard our research on the sensitivity of the episodic buffer to both top-down executive control and bottom-up perceptual input (Baddeley et al., 2017) as giving valuable insight into the way the central executive operates. Figure 1 illustrates the general picture we have developed in light of this work. What is needed now is a plausible way of thinking about the full range of executive functions, one sufficiently simple to be applied to practical problems.

## Conclusions

Our main conclusion is that the multicomponent model continues to meet its original aims. That is, it maintains its robustness in providing a demonstrably useful framework for asking new questions, while at the same time remaining sufficiently simple to be widely applicable. The model has been extended and adjusted over the years to account for an increasing range of phenomena while at the same time retaining its core assumptions. We suggest that its basic distinctions between resources for attentional processing and temporary storage, and between verbal and visuospatial temporary information, are standing the test of time remarkably well. As we have aimed to illustrate, the model’s simplicity allows modifications to be justified and openly evaluated, and its incompleteness allows scope for continuing theoretical development. We began some 50 years ago with the very simple question: *What is short-term memory for?* We continue to hope that our ongoing attempts to find an answer will convince the reader not necessarily that our views are correct but that the question was, and indeed still is, well worth asking.

## Notes

1. We combined proportion correct recognition performance from 5 similar experiments (Allen et al., 2006, Exp. 2–4, and Allen et al., 2012, Exp. 1–2, total *n* = 112), examining proportional change in performance as *(complex task – simple task)/simple task*. Mean proportional change from simple to complex concurrent task conditions was equivalent for colour (−.14), shape (−.14), and binding (−.12), *p* = .37, *BF_10_* = .080. Cowan et al.’s (2024) suggestion that binding information appears to sometimes be proportionally more sensitive to divided attention is based on Hits-False Alarms (H-FA), which was the primary outcome measure originally reported in those studies. However, analysis of proportional change in H-FA at an individual participant level is not appropriate and can produce extreme values in some cases; hence, our use of proportion correct here.
